# Absolute lymphocyte count and neutrophil-to-lymphocyte ratio as predictors of CDK 4/6 inhibitor efficacy in advanced breast cancer

**DOI:** 10.1038/s41598-024-60101-x

**Published:** 2024-04-30

**Authors:** Shogo Nakamoto, Tadahiko Shien, Takayuki Iwamoto, Shinichiro Kubo, Mari Yamamoto, Tetsumasa Yamashita, Chihiro Kuwahara, Masahiko Ikeda

**Affiliations:** 1 Department of Breast and Thyroid Surgery, Fukuyama City Hospital, 5-23-1 Zao, Fukuyama, 721-8511, Japan; 2 Department of Breast and Endocrine Surgery, Okayama University Hospital, 2-5-1, Shikata-Cho, Kita-Ku, Okayama, 700-8558 Japan

**Keywords:** Medical research, Oncology

## Abstract

Cyclin-dependent kinase 4 and 6 inhibitors (CDK4/6i) are the standard agents for treating patients with estrogen receptor-positive and human epidermal growth factor receptor 2-negative advanced breast cancer (ER + HER2 − ABC). However, markers predicting the outcomes of CDK4/6i treatment have yet to be identified. This study was a single-center retrospective cohort study. We retrospectively evaluated 101 patients with ER + HER2 − ABC receiving CDK4/6i in combination with endocrine therapy at Fukuyama City Hospital between November 2017 and July 2021. We investigated the clinical outcomes and the safety of CDK4/6i treatment, and the absolute lymphocyte count (ALC) and neutrophil-to-lymphocyte ratio (NLR) as predictive markers for CDK4/6i. We defined the cut-off values as 1000/μL for ALC and 3 for NLR, and divided into “low” and “high” groups, respectively. We evaluated 43 and 58 patients who received abemaciclib and palbociclib, respectively. Patients with high ALC and low NLR had significantly longer overall survival than those with low ALC and high NLR (high vs. low; ALC: HR 0.29; 95% CI 0.12–0.70; NLR: HR 2.94; 95% CI 1.21–7.13). There was no significant difference in efficacy between abemaciclib and palbociclib and both had good safety profiles. We demonstrated that ALC and NLR might predict the outcomes of CDK4/6i treatment in patients with ER + HER2 − ABC.

## Introduction

Breast cancer, a commonly diagnosed malignancy, is the leading cause of cancer death in women^1^. Advanced breast cancer (ABC) currently remains incurable, with the purpose of treatment being prolongation of survival and maintenance or improvement of quality of life^2^. The majority of patients with ABC have estrogen receptor-positive (ER +) and human epidermal growth factor receptor 2-negative (HER2 −) disease with a relatively indolent course, which is commonly treated with endocrine therapies^2–4^. However, the majority of patients progress during endocrine therapy (acquired resistance), and several patients may fail to respond to initial therapy (de novo resistance)^5^.

Cyclin-dependent kinase (CDK)4 and CDK6 in complex with D-type cyclin catalysts are critical regulators of cell cycle progression^6^, and the CDK4/6/retinoblastoma tumor suppressor protein pathway has important implications in endocrine therapy resistance^7,8^. Thus, targeting CDK4 and CDK6 has been an effective approach for attenuating the growth of ER + breast cancer^6–8^. Recently, several clinical trials have demonstrated the efficacy and safety of CDK4 and CDK6 inhibitors (CDK4/6i) in combination with endocrine therapy for patients with ER + HER2 − ABC^9–14^. In fact, three CDK4/6is have been approved for the treatment of patients with ER + HER2 − ABC, establishing CDK4/6i as the standard for treating ER + HER2 − ABC^3,15,16^. In Japan, abemaciclib (ABM) and palbociclib (PAL) have been approved for use in clinical practice under the Japanese medical insurance system^16^.

The three approved CDK4/6is have different dosage protocols, pharmacokinetics, and target selectivity, despite targeting the same CDK4/6 and having similar clinical indications^17,18^. In particular, ABM targets CDK1-cyclin B and CDK2-cyclin A/E complexes as secondary targets and has a substantially wider range of inhibitory activities than other CDK4/6is^17^. A study demonstrated ABM to be effective against breast cancer cells through various mechanisms, including cell cycle arrest, induction of senescence by prolonged exposure, apoptosis, and alterations in energy metabolism^19^. These mechanisms may be associated with the single-agent activity observed in a clinical trial of ABM^19,20^. In addition, that study showed ABM to promote earlier senescence and apoptosis of hormone receptor-positive breast cancer cells and at lower concentrations than other CDK4/6is^19^. Furthermore, although PAL did not exert efficacy in patients with hormone receptor-positive early breast cancer, ABM did show efficacy in clinical trials^21,22^. Hence, physicians may prefer to select ABM over PAL given the difference in efficacy. However, there are no head-to-head randomized controlled trials directly comparing CDK4/6i, and predictive markers for the outcomes of CDK4/6i treatment remain unclear.

A recent report showed that selective CDK4/6i induces not only tumor cell cycle arrest but also antitumor immunity^23^. Several studies have indicated that systemic immunity markers, including absolute lymphocyte count (ALC) and the neutrophil-to-lymphocyte ratio (NLR), can be used as predictive markers for patients with ABC undergoing eribulin and bevacizumab therapy^24–27^. Therefore, we hypothesized that systemic immunity markers might be useful for predicting responses to CDK4/6i treatment and for determining which agent would be most appropriate for use. We evaluated the correlations between systemic immunity markers and the efficacy of CDK4/6i. In addition, we directly compared the efficacy and safety of ABM and PAL by retrospectively evaluating patients with ER + HER2 − ABC.

## Results

### Patient characteristics

We evaluated 101 ER + HER2 − ABC patients who received CDK4/6i in combination with endocrine agents as first- or second-line therapy, 43 (42.6%) of whom received ABM and 58 (57.4%) PAL.

The patient characteristics at baseline are shown in Table 1. The median ages were 60 years (range 29–84 years) and 64 years (range 38–100 years) in the ABM and PAL groups, respectively. Compared to the ABM group, the PAL group included more postmenopausal women (57.1% vs. 81.0%, *P* = 0.014), patients who received CDK4/6i as second-line therapy (18.6% vs. 51.7%, *P* = 0.001), those given letrozole as endocrine agents (2.3% vs. 27.6%, *P* = 0.001), and patients requiring CDK4/6i with dose reduction at the start of administration (4.7% vs. 36.2%, *P* < 0.001).

**Table 1:** Patient characteristics at baseline.

Variables	Abemaciclib	Palbociclib	*P*
	N = 43 (%)	N = 58 (%)	
Age, median, years (range)	60 (29–84)	64 (38–100)	0.064
Menopausal status^a^			0.014
Premenopausal	18 (42.9)	11 (19.0)	
Postmenopausal	24 (57.1)	47 (81.0)	
Diagnosis			0.84
De novo	21 (48.8)	26 (44.8)	
Recurrence	22 (51.2)	32 (55.2)	
Disease-free interval^b^			0.56
< 24 months	2 (9.1)	1 (3.2)	
≥ 24 months	20 (90.9)	30 (96.8)	
Prior (neo)adjuvant chemotherapy^c^			0.84
Yes	15 (34.9)	19 (32.8)	
No	28 (65.1)	39 (67.2)	
Endocrine sensitivity^d^			0.29
Sensitive	32 (74.4)	37 (63.8)	
Resistant	11 (25.6)	21 (36.2)	
Metastatic sites			
Central nervous system	3 (7.0)	2 (3.4)	0.65
Bone	30 (69.8)	38 (65.5)	0.68
Lungs	18 (41.9)	20 (34.5)	0.53
Pleura and/or lymphangiopathy	30 (69.8)	43 (74.1)	0.66
Lymph nodes	11 (25.6)	16 (27.6)	1.00
Liver	10 (23.3)	18 (31.0)	0.50
Soft tissue^e^	22 (51.2)	34 (58.6)	0.55
Type of metastases			0.84
Visceral	26 (60.5)	33 (56.9)	
Non-visceral	17 (39.5)	25 (43.1)	
Number of metastatic sites			0.84
≥ 3	16 (37.2)	23 (39.7)	
< 3	27 (62.8)	35 (60.3)	
Prior chemotherapy^f^			0.84
Yes	15 (34.9)	19 (32.8)	
No	28 (65.1)	39 (67.2)	
CDK4/6i treatment line			0.001
First	35 (81.4)	28 (48.3)	
Second	8 (18.6)	30 (51.7)	
Endocrine agents			0.001
Letrozol	1 (2.3)	16 (27.6)	
Fulvestrant	42 (97.7)	42 (72.4)	
Dose reduction at the start of administration			< 0.001
Yes	2 (4.7)	21 (36.2)	
No	41 (95.3)	37 (63.8)	

CDK4/6i, cyclin-dependent kinase 4 and 6 inhibitors.

^a^One patient was male in the abemaciclib group.

^b^We excluded de novo breast cancer.

^c^(Neo)adjuvant chemotherapy included anthracycline and/or taxane-based regimens.

^d^Endocrine resistance was defined as recurrence during adjuvant endocrine therapy.

^e^Soft tissue included the contralateral breast, muscle, and skin.

^f^Chemotherapy for advanced breast cancer.

### Correlations between systemic immunity markers and the efficacy of CDK4/6i

**Figure 1:**
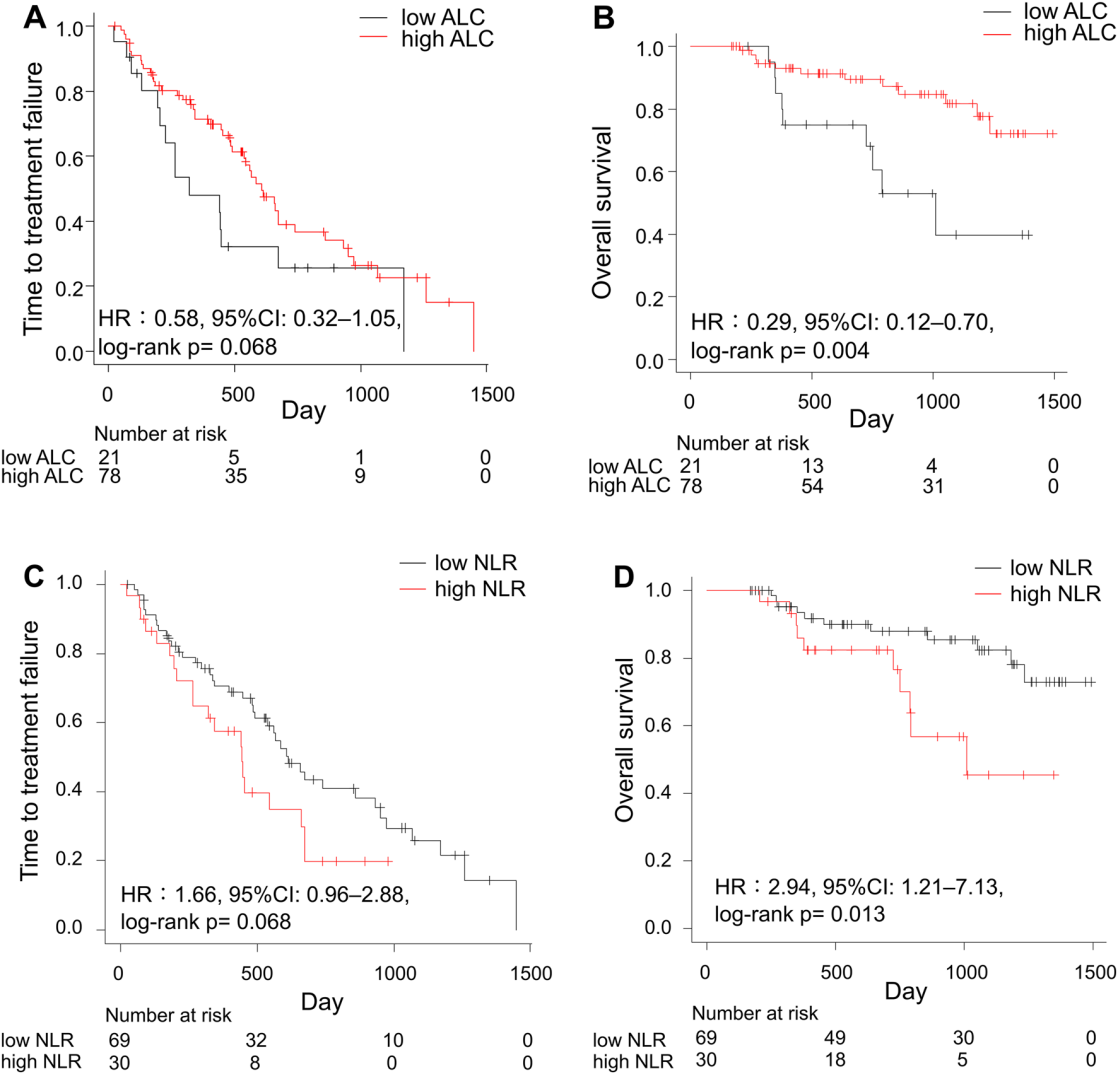
Time to treatment failure and overall survival according to baseline values of (**A**,**B**) ALC and (**C**,**D**) NLR in patients treated with CDK4/6i for advanced breast cancer. *ALC* absolute lymphocyte count, *CDK4/6i* cyclin-dependent kinase 4 and 6 inhibitors, *CI* confidence interval, *HR* hazard ratio, *NLR* neutrophil-to-lymphocyte ratio.

The data acquisition cut-off was set at February 2022, and the median follow-up duration was 751 days. Time to treatment failure (TTF) and overall survival (OS) were compared according to systemic immunity markers (Fig. 1). We excluded one patient in the ABM and one in the PAL group due to missing systemic immunity marker data. Although not significant, patients with high ALC and low NLR tended to have longer TTF than those with low ALC and high NLR [high vs. low; ALC: 609 vs. 322 days; hazard ratio (HR), 0.58; 95% confidence interval (CI) 0.32–1.05; log-rank *P* = 0.068; high vs. low; NLR: 443 vs. 615 days; HR 1.66; 95% CI 0.96–2.88; log-rank *P* = 0.068; Fig. 1A,C]. Patients with high ALC and low NLR had significantly higher 2-year OS than those with low ALC and high NLR (high vs. low; ALC: 89.4% vs. 68.2%; HR, 0.29; 95% CI 0.12–0.70; log-rank *P* = 0.004; high vs. low; NLR: 76.6% vs. 87.9%; HR 2.94; 95% CI 1.21–7.13; log-rank *P* = 0.013; Fig. 1B,D).

**Figure 2:**
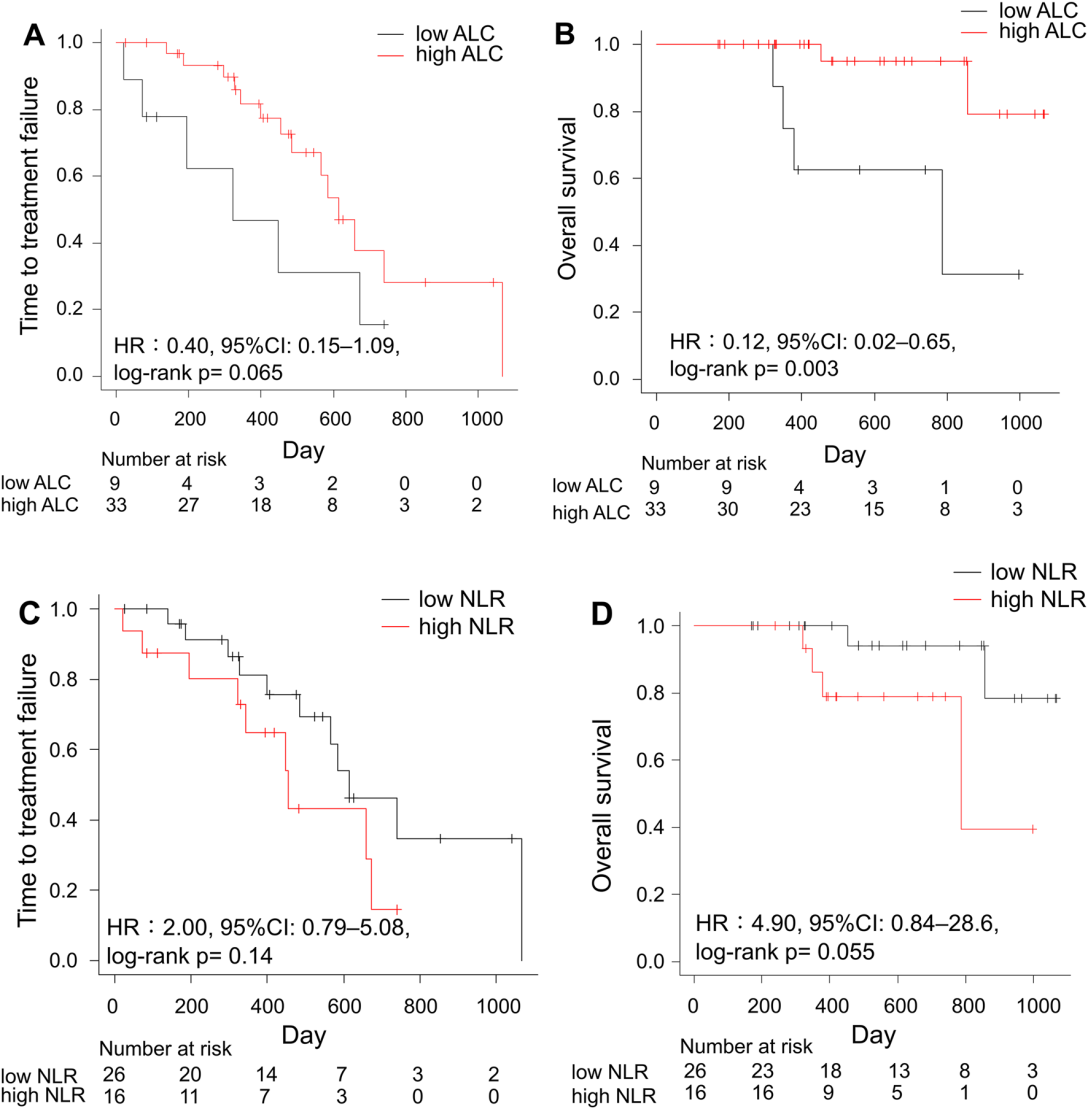
Time to treatment failure and overall survival according to baseline values of (**A**,**B**) ALC and (**C**,**D**) NLR in patients treated with ABM for advanced breast cancer. ABM, abemaciclib; *ALC* absolute lymphocyte count, *CI* confidence interval, *HR* hazard ratio, *NLR* neutrophil-to-lymphocyte ratio.

In addition, we examined the usefulness of systemic immunity markers as predicters in the ABM and PAL groups (Fig. 2 and Supplementary Fig. 1). No difference in TTF was observed according to ALC and NLR values (high or low) in either the ABM (Fig. 2A,C) or the PAL group (Supplementary Fig. 1A,C). Among patients in the ABM group, those with high ALC had significantly higher 2-year OS than those with low ALC (95.0% vs. 62.5%; HR 0.12; 95% CI 0.02–0.65; log-rank *P* = 0.003, Fig. 2B). However, no difference was observed in the PAL group (85.8% vs. 72.9%; HR 0.44; 95% CI 0.15–1.31; log-rank *P* = 0.13, Supplementary Fig. 1B). We conducted an interaction test to assess the association between ALC and the predictive benefit of each CDK4/6i. No significant interaction was observed (p = 0.22).

We performed univariate and multivariate analyses to determine the association between baseline patient characteristics and TTF and OS and evaluated the utility of systemic immunity markers as predictors of outcomes (Table 2). Each of the two multivariate analyses indicated that ALC and NLR were not independently associated with longer TTF (high vs. low; ALC: HR 0.64; 95% CI 0.38–1.09; *P* = 0.10; NLR: HR 1.41; 95% CI 0.78–2.56; *P* = 0.26). However, high ALC and low NLR were independently associated with longer OS (high vs. low; HR 0.10; 95% CI 0.02–0.58; *P* = 0.011; NLR: HR 5.17; 95% CI 1.20–22.3; *P* = 0.028). The multivariate Cox regression analysis results are shown in Supplementary Tables S1 and S2.

**Table 2:** Univariate analysis of time to treatment failure and overall survival (Cox hazard model).

Variables	TTF			OS		
	HR	95% CI	*P*	HR	95% CI	*P*
Age	0.99	0.97–1.01	0.32	1.00	0.96–1.04	0.91
Menopausal status^a^ (pre- vs. postmenopausal)	0.77	0.41–1.42	0.40	0.99	0.36–2.69	0.98
Diagnosis (recurrence vs. de novo)	1.06	0.63–1.77	0.83	0.90	0.39–2.09	0.81
Disease-free interval^b^ (< 24 vs. ≥ 24 months)	2.00	0.46–8.60	0.35	6.81	1.24–37.5	0.028
Prior (neo)adjuvant chemotherapy^c^ (yes vs. no)	1.44	0.85–2.42	0.17	1.05	0.44–2.50	0.92
Endocrine sensitivity^d^ (sensitive vs. resistant)	1.35	0.79–2.30	0.27	0.99	0.40–2.46	0.99
Metastatic sites (yes vs. no)						
Central nervous system	3.56	1.24–10.3	0.019	4.37	1.27–15.0	0.019
Bone	0.85	0.50–1.43	0.53	1.43	0.56–3.65	0.46
Lungs	1.05	0.63–2.12	0.86	1.26	0.55–2.93	0.59
Pleura and/or lymphangiopathy	1.61	0.94–2.76	0.085	1.10	0.45–2.70	0.84
Lymph nodes	1.18	0.66–2.12	0.58	0.40	0.17–0.94	0.035
Liver	1.39	0.80–2.41	0.25	1.39	0.80–2.41	0.25
Soft tissue^e^	0.89	0.53–1.48	0.64	0.65	0.28–1.52	0.32
Visceral metastasis (yes vs. no)	1.36	0.80–2.31	0.26	1.78	0.69–4.53	0.23
Number of metastatic sites (≥ 3 vs. < 3)	1.11	0.66–1.87	0.69	1.29	0.53–3.18	0.58
Prior chemotherapy^f^ (yes vs. no)	1.96	1.16–3.31	0.012	2.49	1.07–5.76	0.034
CDK4/6i agents (PAL vs. ABM)	1.07	0.62–1.86	0.81	0.79	0.31–2.05	0.63
Endocrine agents (LET vs. FUL)	1.27	0.70–2.32	0.43	0.89	0.32–2.44	0.82
Dose reduction at the start of administration (yes vs. no)	0.91	0.48–1.71	0.76	1.01	0.37–2.75	0.98
Marker of systemic immunity at baseline						
ALC > 1000 vs. ALC ≤ 1000	0.58	0.32–1.05	0.072	0.29	0.12–0.70	0.006
NLR > 3 vs. NLR ≤ 3	1.66	0.96–2.88	0.072	2.94	1.21–7.13	0.017

*ABM* abemaciclib, *ALC* absolute lymphocyte count, *CI* confidence interval, *CDK4/6i* cyclin-dependent kinases 4 and 6 inhibitors, *HR* hazard ratio, *OS* overall survival, *PAL* Palbociclib, *TTF* time to treatment failure.

^a^One patient was male in the abemaciclib group.

^b^We excluded de novo breast cancer.

^c^(Neo)adjuvant chemotherapy included anthracycline and/or taxane-based regimens.

^d^Endocrine resistance was defined as recurrence during adjuvant endocrine therapy.

^e^Soft tissue included the contralateral breast, muscle, and skin.

^f^Chemotherapy for advanced breast cancer.

### Comparison of efficacy between ABM and PAL

**Figure 3:**
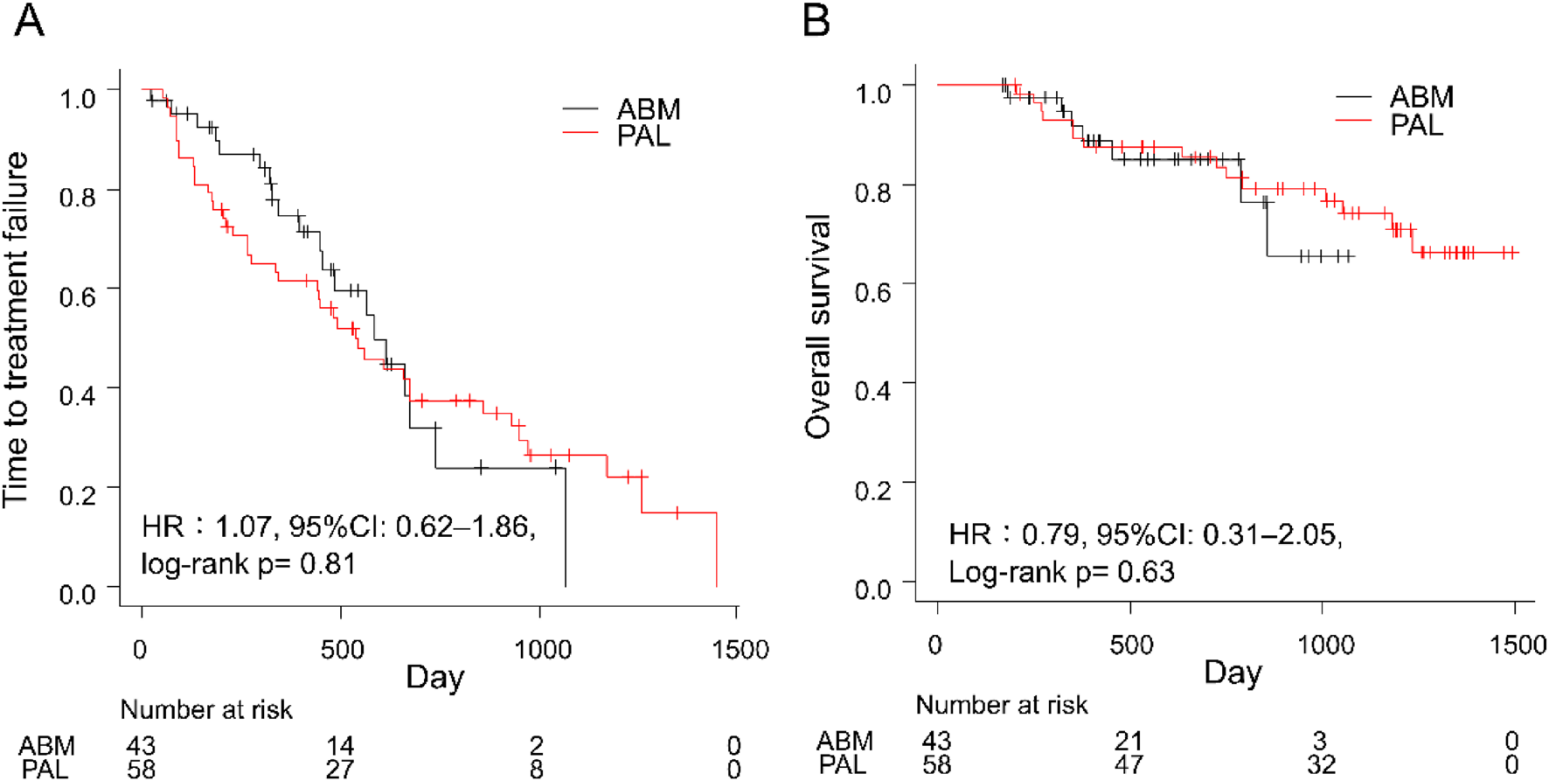
Time to treatment failure (**A**) and overall survival (**B**) according to CDK4/6i in patients with advanced breast cancer. *ABM* abemaciclib, *CDK4/6i* cyclin-dependent kinase 4 and 6 inhibitors, *CI* confidence interval, *HR* hazard ratio, *PAL* palbociclib.

In directly comparing the efficacy of ABM and PAL, we found no significant difference in the TTF and 2-year OS between these two groups (Fig. 3). The TTF were 585 days (95% CI 447–741 days) and 539 days (95% CI 336–861 days) in the ABM and PAL groups, respectively (HR 1.07; 95% CI 0.62–1.86, log-rank *P* = 0.81; Fig. 3A). The 2-year OS were 85.0% (95% CI 67.2%–93.5%) and 83.5% (95% CI 70.5%–91.0%) in the ABM and PAL groups, respectively (HR 0.79; 95% CI 0.31–2.05, log-rank *P* = 0.63; Fig. 3B).

Comparing the outcomes according to treatment lines revealed no significant difference in either TTF or 2-year OS between the two groups in patients who received CDK4/6i as first-line treatment (ABM vs. PAL; TTF: 567 vs. 861 days; HR 0.75; 95% CI 0.37–1.55; log-rank *P* = 0.44; 2-year OS: 79.9% vs. 88.4%; HR 0.59; 95% CI 0.18–1.95; log-rank *P* = 0.38; Supplementary Fig. 2A,B). Similar results were obtained in patients who received CDK4/6i as second-line treatment (ABM vs. PAL; TTF: 741 vs. 483 days; HR 2.00; 95% CI 0.69–5.84; log-rank *P* = 0.20; 2-year OS: 100% vs. 78.6%; HR 1.63; 95% CI 0.20–13.5; log-rank *P* = 0.65; Supplementary Fig. 2C,D).

### Comparison of safety between ABM and PAL

In the population assessed for safety (n = 43 in the ABM group; n = 58 in the PAL arm), the most frequent adverse events of any grade in the ABM group were diarrhea/constipation, fatigue, and neutropenia. In contrast, the most frequent adverse events of any grade in the PAL group were neutropenia, anemia, and fatigue (Table 3). Grade 3/4 adverse events, mostly neutropenia, occurred more frequently in the PAL group than in the ABM group (37.2% vs. 82.8%, respectively).

**Table 3:** Adverse events.

Variables	Abemaciclib N = 43 (%)		Palbociclib N = 58 (%)		P*	P**
	Any grade	Grade 3/4	Any grade	Grade 3/4		
Any adverse events	43 (100)	16 (37.2)	58 (100)	48 (82.8)	*NA*	< 0.001
Neutropenia	34 (79.1)	13 (30.2)	58 (100)	46 (79.3)	< 0.001	< 0.001
Anemia	33 (76.7)	4 (9.3)	48 (82.8)	6 (10.3)	0.46	1.00
Platelet count decreased	23 (53.5)	0 (0)	31 (53.4)	2 (3.4)	1.00	0.51
Nausea/vomiting	15 (34.9)	1 (2.3)	18 (31.0)	0 (0)	0.83	0.43
Decreased appetite	28 (65.1)	0 (0)	29 (50.0)	0 (0)	0.16	*NA*
Oral mucositis	14 (32.6)	0 (0)	27 (46.6)	0 (0)	0.22	*NA*
Fatigue	34 (79.1)	0 (0)	41 (70.7)	0 (0)	0.37	*NA*
Diarrhea/constipation	40 (93.0)	0 (0)	34 (58.6)	0 (0)	< 0.001	*NA*
AST/ALT increased	29 (67.4)	0 (0)	21 (36.2)	0 (0)	0.003	*NA*
Interstitial pneumonia	8 (18.6)	0 (0)	3 (5.2)	0 (0)	0.050	*NA*
Rash or desquamation	1 (2.3)	0 (0)	8 (13.8)	0 (0)	0.074	*NA*
Alopecia	4 (9.3)	0 (0)	1 (1.7)	0 (0)	0.16	*NA*
Dysgeusia	16 (37.2)	0 (0)	12 (20.7)	0 (0)	0.076	*NA*

*AST* aspartate aminotransferase level, *ALT* alanine aminotransferase, *NA* not applicable.

*Comparison of any grade adverse events.

**Comparison of grade 3/4 adverse events.

There was a significant difference in the incidence of adverse events between the two groups. Grade 3/4 neutropenia was observed in 30.2% and 79.3% of patients in the ABM and PAL groups, respectively (*P* < 0.001). Diarrhea and constipation occurred in 93.0% and 58.6% of the patients in the ABM and PAL groups, respectively (*P* < 0.001). Laboratory-based abnormalities, such as increased aspartate aminotransferase/alanine aminotransferase levels, were observed in 67.4% and 36.2% of the ABM and PAL group patients, respectively (*P* = 0.003). Interstitial pneumonia occurred in 18.6% and 5.2% of patients in the ABM and PAL groups, respectively (*P* = 0.050). No new safety concerns associated with the therapy were identified.

## Discussion

Our study demonstrated ALC and NLR to be significantly associated with longer OS in patients with ER + HER2 − ABC who received CDK4/6i, and both were useful as markers for predicting the outcomes of CDK4/6i treatment. In addition, the current study directly compared the efficacy and safety of ABM and PAL, and showed no significant difference in efficacy between the ABM and PAL groups. The safety profile of each therapy was acceptable, with treatments being well tolerated in patients with ER + HER2 − ABC who received CDK4/6i.

Several studies have demonstrated systemic immunity markers such as ALC and NLR to be useful as prognostic markers in breast cancer and other malignant tumors^28–32^. In addition, numerous studies have shown that systemic immunity markers are also useful for predicting the results obtained with certain treatment regimens in patients with ABC^24–27^. High ALC and low NLR are reportedly associated with improved progression-free survival and OS in patients receiving eribulin and paclitaxel plus bevacizumab^24–27^. These results suggested that systemic immunity markers may predict the systemic antitumor activity promoted by treatment, thereby possibly enhancing the antitumor immune response in patients with ABC^24–27^.

A recent report showed that selective CDK4/6i induced not only tumor cell cycle arrest but also antitumor immunity^23^. CDK4/6i activates tumor cell expression of endogenous retroviral elements and increases intracellular levels of double-stranded RNA. These responses in turn stimulate the production of type III interferons and enhance the presentation of tumor antigens. CDK4/6i significantly suppresses the proliferation of regulatory T cells. Furthermore, the addition of immune checkpoint blockade agents reportedly enhanced antitumor immunity^23^. Therefore, we hypothesized that systemic immunity markers may be associated with the efficacy of CDK4/6i and might thus serve as markers for determining which CDK4/6i would be most appropriate for administration. Our results showed that patients with high ALC and low NLR had significantly better 2-year OS than those with low ALC and high NLR, suggesting the usefulness of ALC and NLR as markers for predicting the outcomes of CDK4/6i treatment. In addition, we examined the usefulness of systemic immunity markers in determining which CDK4/6i should be selected. Although patients with high ALC had significantly better 2-year OS than those with low ALC in the ABM group, no difference was observed in the PAL group. However, the interaction test did not show any statistically significant differences. Therefore, ALC may have potential utility in the selection of CDK4/6i, but caution is warranted in interpreting this due to the single-center retrospective nature of the study.

Several studies indirectly comparing CDK4/6i have shown the efficacy of PAL to be comparable to that of ABM and ribociclib (RIB)^33,34^. A network meta-analysis, which included a total of 11 randomized controlled trials including 4178 patients, showed that PAL did not significantly prolong PFS compared to ABM (HR 0.83, 95% credible interval: 0.60–1.16)^33^. A study that aimed to determine the relative efficacy of PAL and compare it to those of RIB and ABM using matching-adjusted indirect treatment comparisons showed that PAL achieved an OS similar to those obtained with ABM (HR 0.87; 95% CI 0.54–1.40) and RIB (HR 0.89; 95% CI 0.48–1.63)^34^. Given the lack of head-to-head randomized controlled trials directly comparing CDK4/6i, we conducted a direct comparison of the efficacy and safety of ABM and PAL using real-world data. Despite the differences in patient backgrounds between the two groups, our findings showed no significant differences in efficacy between ABM and PAL, corroborating most of the prior results of indirect treatment comparisons^33,34^.

Consistent with clinical trial results^9–12^, our findings revealed differences in the incidence of adverse events between ABM and PAL. A study using anchored matching-adjusted indirect comparison methods leveraging individual patient data from clinical studies^9,12^ showed PAL to be associated with significantly greater improvements than ABM across several symptom subscales, including nausea/vomiting, appetite loss, diarrhea, and systemic therapy side effects^35^. In addition, our results showed that interstitial pneumonia occurred more frequently in the ABM group (18.6%) than in the PAL group (5.2%; *P* = 0.050). A study that assessed the pulmonary toxicity of CDK4/6i by analyzing the publicly available FDA Adverse Event Reporting System demonstrated that interstitial lung disease represented 2.1% of total reports recorded for ABM but only 0.3% of total reports recorded for PAL and RIB^36^. Therefore, in certain patients, particularly those with a history of lung disease, PAL might be preferable to ABM in terms of the associated adverse events^35,36^. Given that CDK4/6i have different safety profiles, physicians need to optimize their use of these agents according to differences in adverse events and patient preferences.

Our study has limitations due to its retrospective nature. First, the number of cases was small, and unexpected biases were present. Since this is a retrospective study, we did not set a sample size and examined the cases we were able to collect as feasibly as possible. However, we believe that the sample size of our study was adequate for meaningful analysis and interpretation because previous reports have been validated and concluded with 94–144 patients^24,26,27^. Second, caution is necessary when interpreting the results of endocrine susceptibility given that our definition was not based on clinical studies^9,12^. However, considering our use of real-world data, the strengths of our study are its high external validity and that we were able to directly compare ABM and PAL, which previous clinical trials failed to do. In addition, a multicenter prospective study directly comparing the efficacy and safety of ABM and PAL in Japan is currently ongoing (UMIN000035533), and the results are anticipated to be of particular interest. Third, the precise mechanisms underlying how ALC and NLR act as predictive factors for the therapeutic efficacy of CDK4/6i remain unclear. It is possible that ALC and NLR merely reflect a favorable immune status as systemic immune markers. Basic research is needed to clarify how patients' ALC and NLR levels alter in response to treatment. Furthermore, the impact of dynamic changes in ALC and NLR following CDK4/6i therapy on treatment outcomes is uncertain, highlighting the need for further investigation.

In conclusion, our study demonstrated ALC and NLR to be significantly associated with longer OS in patients with ER + HER2 − ABC who received CDK4/6i, and might thus be useful as markers for predicting the outcomes of CDK4/6i treatment. Our findings, alongside those of Gerratana et al., underscore the need for further exploration into the prognostic value of systemic immunity markers in breast cancer^37,38^. In addition, we directly compared the efficacy and safety of ABM and PAL, and found no significant efficacy difference between the ABM and PAL groups. The safety profiles of both therapies were good, with each being well tolerated by patients with ER + HER2 − ABC who received CDK4/6i. Given the similar efficacies of ABM and PAL, physicians should select the optimal CDK4/6i treatment based on patient preference and adverse event profiles.

## Methods

### Patients and treatments

Patients with ER + HER2 − ABC who received CDK4/6i combined with endocrine therapy as first/second-line treatment at Fukuyama City Hospital between November 2017 and July 2021 were retrospectively evaluated. We enrolled patients who underwent CDK4/6i therapy as either first or second-line endocrine treatment for ABC. Patients who received CDK4/6i therapy as third or later-line endocrine treatment for ABC, those who received it as adjuvant endocrine therapy for early breast cancer, or those without whole blood samples were excluded from the study. Their medical records were reviewed to determine patient backgrounds and outcomes. Pathological reports of surgical specimens or initial biopsy specimens were used. We also preferentially used biopsies from metastases and recurrent tumors when available. We defined ABC as locally advanced and/or metastatic breast cancer^2^. ER positivity was defined as ≥ 1% positivity for ER. HER2 negativity was defined as immunohistochemistry 1 + or 0, or negative in situ hybridization, following the guidelines of the American Society of Clinical Oncology/College of American Pathologists guidelines^39^. Clinical responses were evaluated according to the Response Evaluation Criteria in Solid Tumors version 1.1^40^.

The CDK4/6i, and the endocrine therapy to be used in combination with CDK4/6i, as well as the sequencing treatment regimens were chosen based on the guidelines^3,15,16^ and shared decision making between physicians and patients as in routine clinical practice. Dose modifications, interruptions, and discontinuations were also determined according to routine clinical practice.

All procedures involving human participants were performed in accordance with the ethical standards of the institutional and/or national research committee and with the 1964 Declaration of Helsinki and its later amendments or comparable ethical standards. This study was conducted in full compliance with the law and after approval had been obtained from the Fukuyama Municipal Hospital Institutional Review Board (approval number: 595). Due to the retrospective nature of the study, the need of informed consent was waived by Fukuyama Municipal Hospital Ethics review committee.

### Measurements of systemic immune markers

Whole blood samples were obtained from ABC patients at or before administering CDK4/6i treatment, and neutrophil and lymphocyte counts were measured using a Sysmex XE-2100 or XE-5000 automated hematology system (Sysmex Co., Kobe, Japan)^26,27^. The ALC and NLR were calculated from blood cell counts, and the cut-off values of these parameters were defined according to previous studies^24–27^: 1000/μL for ALC and 3 for NLR. All patients were divided into “low” and “high” groups based on the cut-off values^24–27^.

### Statistical analysis

Wilcoxon’s rank sum test was used to compare continuous variables, whereas Fisher’s exact tests were applied to compare categorical variables between groups. Survivals were estimated using the Kaplan–Meier method and then compared using the log-rank test. Cox regression models were used for univariate and multivariate analyses. Covariates with a *P* value < 0.10 during univariate analysis were included in the multivariate analysis. Given the correlation between ALC and NLR, we did not include these markers simultaneously during multivariate analysis; however, we included these markers independently in each multivariate analysis of TTF and OS^26,27^. In all statistical analyses, *P* < 0.05 was considered to indicate a significant result. All analyses were performed using EZR software (Saitama Medical Center, Jichi Medical University, Saitama, Japan), a graphical user interface for R (The R Foundation for Statistical Computing, Vienna, Austria)^41^.

This study defined endocrine resistance as recurrence during adjuvant endocrine therapy. TTF was defined as the duration from the administration of CDK4/6i in combination with endocrine therapy for ER + HER2 − ABC to the discontinuation of treatment for any reason, including disease progression, treatment-induced toxicity, patient/physician choice, and death from any cause. OS was defined as the duration from the administration of CDK4/6i in combination with endocrine therapy to the date of death from any cause. Due to the short observation period in this study, we also evaluated the 2-year OS.

### Supplementary Information

Supplementary Information.

## Data Availability

The datasets generated during and/or analyzed during the current study are available from the corresponding author on reasonable request.
