# Supplementary material for: Absolute lymphocyte count and neutrophil-to-lymphocyte ratio as predictors of CDK 4/6 inhibitor efficacy in advanced breast cancer
Source: Sci Rep. 2024 Apr 30;14:9869. doi: 10.1038/s41598-024-60101-x (PMC11059159; doi:10.1038/s41598-024-60101-x)
Supplement: Supplementary file 1 — Supplementary Information. [file 41598_2024_60101_MOESM1_ESM.docx]

**[Manuscript title]**

Absolute Lymphocyte Count and Neutrophil-to-Lymphocyte Ratio as Predictors of CDK 4/6 Inhibitor Efficacy in Advanced Breast Cancer

**[Authors and affiliations]**

Shogo Nakamoto^1,2*^, Tadahiko Shien^2^, Takayuki Iwamoto^2^, Shinichiro Kubo^1^, Mari Yamamoto^1^, Tetsumasa Yamashita^1^, Chihiro Kuwahara^1^, and Masahiko Ikeda^1^

1: Department of Breast and Thyroid Surgery, Fukuyama City Hospital, Hiroshima, Japan

2: Department of Breast and Endocrine Surgery, Okayama University Hospital, Okayama, Japan

**[Corresponding author]**

Shogo Nakamoto MD

Department of Breast and Endocrine Surgery, Okayama University Hospital, Japan

2-5-1, Shikata-cho, Kita-ku, Okayama 700-8558, Japan

Phone: +81-86-235-6502

E-mail: [p92c9f20@s.okayama-u.ac.jp](mailto:p92c9f20@s.okayama-u.ac.jp)

Supplementary Table 1. Multivariable analysis of time to treatment failure (Cox hazard model)

| Variables | ALC | | | NLR | | |
| --- | --- | --- | --- | --- | --- | --- |
|  | HR | 95% CI | *P* | HR | 95% CI | *P* |
| Age |  |  |  |  |  |  |
| Menopausal status ^a^ (pre- vs. postmenopausal) |  |  |  |  |  |  |
| Diagnosis (recurrence vs. de novo) |  |  |  |  |  |  |
| Disease-free interval ^b^ (<24 vs. ≥24 months) |  |  |  |  |  |  |
| Prior (neo)adjuvant chemotherapy ^c^ (yes vs. no) |  |  |  |  |  |  |
| Endocrine sensitivity ^d^ (sensitive vs. resistant) |  |  |  |  |  |  |
| Metastatic sites (yes vs. no) |  |  |  |  |  |  |
| Central nervous system | 3.00 | 0.99–9.09 | 0.052 | 2.68 | 0.87–8.22 | 0.085 |
| Bone |  |  |  |  |  |  |
| Lungs |  |  |  |  |  |  |
| Pleura and/or lymphangiopathy | 1.15 | 0.61–2.13 | 0.67 | 1.21 | 0.64–2.27 | 0.55 |
| Lymph nodes |  |  |  |  |  |  |
| Liver |  |  |  |  |  |  |
| Soft tissue ^e^ |  |  |  |  |  |  |
| Visceral metastasis (yes vs. no) |  |  |  |  |  |  |
| Number of metastatic sites (≥3 vs. <3) |  |  |  |  |  |  |
| Prior chemotherapy ^f^ (yes vs. no) | 1.55 | 0.82–2.95 | 0.18 | 1.56 | 0.84–2.90 | 0.16 |
| CDK4/6i agents (PAL vs. ABM) | 1.09 | 0.61–1.93 | 0.77 | 1.12 | 0.63–2.00 | 0.69 |
| Endocrine agents (LET vs. FUL) |  |  |  |  |  |  |
| Dose reduction at the start of administration (yes vs. no) |  |  |  |  |  |  |
| Markers of systemic immunity at baseline |  |  |  |  |  |  |
| ALC >1000 vs. ALC ≤1000 | 0.64 | 0.38–1.09 | 0.10 |  |  |  |
| NLR >3 vs. NLR ≤3 |  |  |  | 1.41 | 0.78–2.56 | 0.26 |

a: one patient was male in the abemaciclib group.

b: we excluded de novo breast cancer.

c: (neo)adjuvant chemotherapy included anthracycline and/or taxane-based regimens.

d: endocrine resistance was defined as recurrence during adjuvant endocrine therapy.

e: soft tissue included the contralateral breast, muscle, and skin.

f: chemotherapy for advanced breast cancer.

Abbreviations: ABM, Abemaciclib; ALC, absolute lymphocyte count; CI, confidence interval; CDK4/6i, Cyclin-dependent kinase 4 and 6 inhibitors; HR, hazard ratio; OS, overall survival; PAL, Palbociclib; TTF, time to treatment failure

Supplementary Table 2. Multivariable analysis of overall survival (Cox hazard model)

| Variables | ALC | | | NLR | | |
| --- | --- | --- | --- | --- | --- | --- |
|  | HR | 95% CI | *P* | HR | 95% CI | *P* |
| Age |  |  |  |  |  |  |
| Menopausal status ^a^ (pre- vs. postmenopausal) |  |  |  |  |  |  |
| Diagnosis (recurrence vs. de novo) |  |  |  |  |  |  |
| Disease-free interval ^b^ (<24 vs. ≥24 months) | 2.24 | 0.27–18.5 | 0.45 | 14.7 | 2.21–98.1 | 0.005 |
| Prior (neo)adjuvant chemotherapy ^c^ (yes vs. no) |  |  |  |  |  |  |
| Endocrine sensitivity ^d^ (sensitive vs. resistant) |  |  |  |  |  |  |
| Metastatic sites (yes vs. no) |  |  |  |  |  |  |
| Central nervous system | NA | NA | NA | NA | NA | NA |
| Bone |  |  |  |  |  |  |
| Lungs |  |  |  |  |  |  |
| Pleura and/or lymphangiopathy |  |  |  |  |  |  |
| Lymph nodes | 0.31 | 0.08–1.28 | 0.11 | 0.30 | 0.07–1.34 | 0.11 |
| Liver |  |  |  |  |  |  |
| Soft tissue ^e^ |  |  |  |  |  |  |
| Visceral metastasis (yes vs. no) |  |  |  |  |  |  |
| Number of metastatic sites (≥3 vs. <3) |  |  |  |  |  |  |
| Prior chemotherapy ^f^ (yes vs. no) | 0.69 | 0.12–3.89 | 0.68 | 1.65 | 0.39–6.96 | 0.50 |
| CDK4/6i agents (PAL vs. ABM) | 0.98 | 0.25–3.89 | 0.98 | 1.51 | 0.34–6.63 | 0.59 |
| Endocrine agents (LET vs. FUL) |  |  |  |  |  |  |
| Dose reduction at the start of administration (yes vs. no) |  |  |  |  |  |  |
| Markers of systemic immunity at baseline |  |  |  |  |  |  |
| ALC >1000 vs. ALC ≤1000 | 0.10 | 0.02–0.58 | 0.011 |  |  |  |
| NLR >3 vs. NLR ≤3 |  |  |  | 5.17 | 1.20–22.3 | 0.028 |

a: one patient was male in the abemaciclib group.

b: we excluded de novo breast cancer.

c: (neo)adjuvant chemotherapy included anthracycline and/or taxane-based regimens.

d: endocrine resistance was defined as recurrence during adjuvant endocrine therapy.

e: soft tissue included the contralateral breast, muscle, and skin.

f: chemotherapy for advanced breast cancer.

Abbreviations: ABM, abemaciclib; ALC, absolute lymphocyte count; CI, confidence interval; CDK4/6i, Cyclin-dependent kinase 4 and 6 inhibitors; HR, hazard ratio; OS, overall survival; PAL, palbociclib; TTF, time to treatment failure.

Supplementary Figure. 1 Time to treatment failure and overall survival according to baseline values of (A, B) ALC and (C, D) NLR in patients treated with PAL for advanced breast cancer


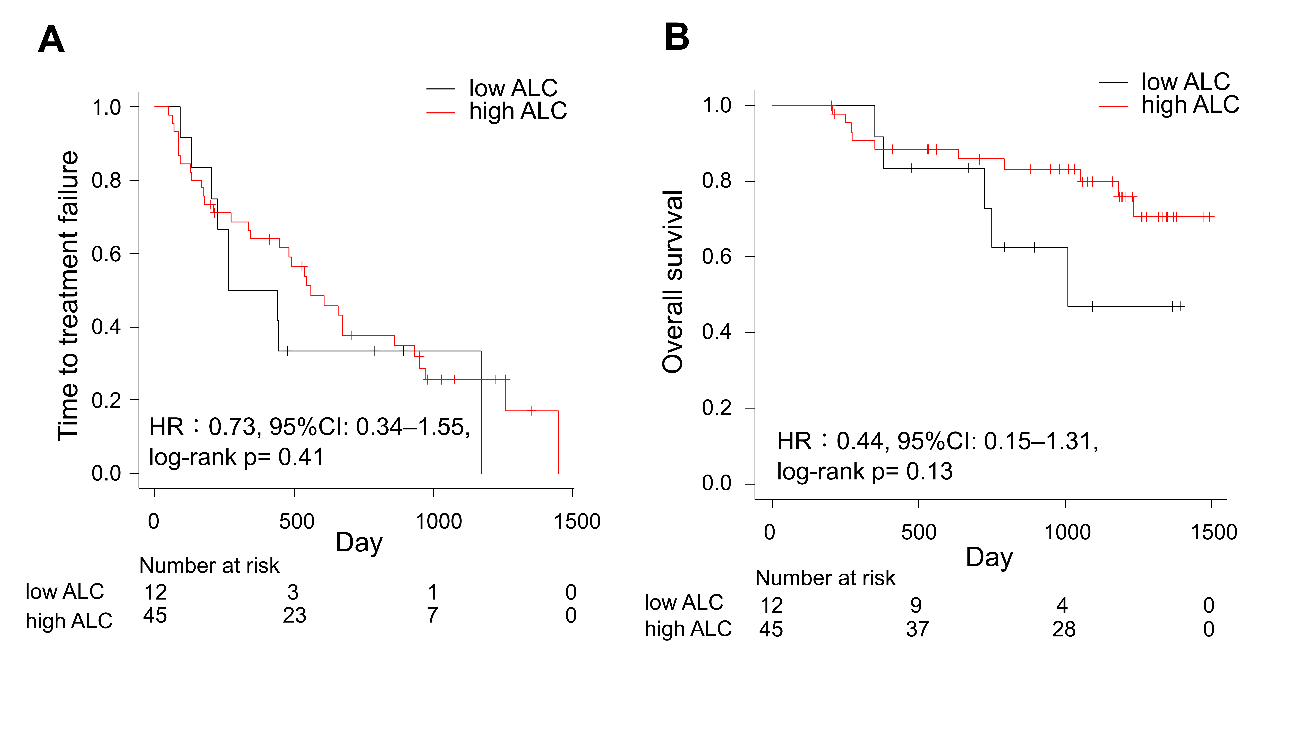


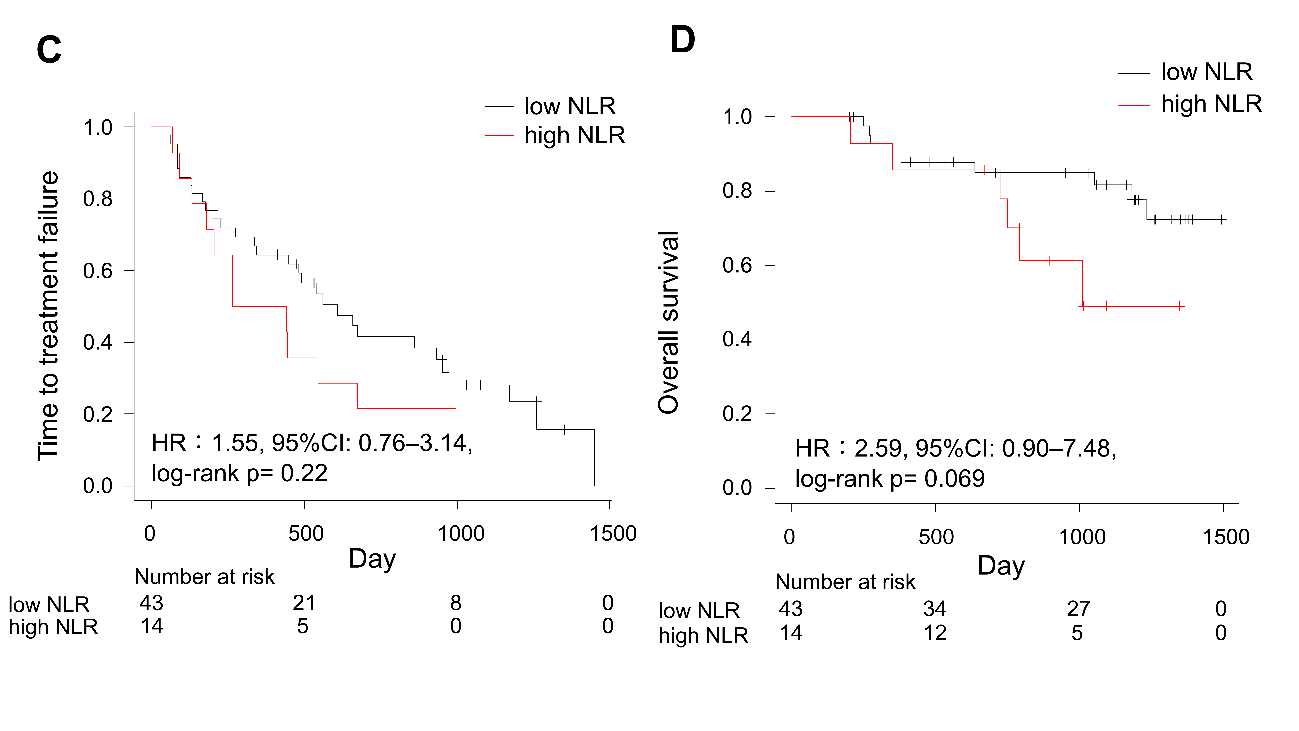


Abbreviations: ALC, absolute lymphocyte count; CI, confidence interval; HR, hazard ratio; NLR, neutrophil-to-lymphocyte ratio; PAL, palbociclib

Supplementary Figure. 2 Time to treatment failure and overall survival in patients treated with CDK4/6i as (A, B) first line and (C, D) second-line therapy for advanced breast cancer


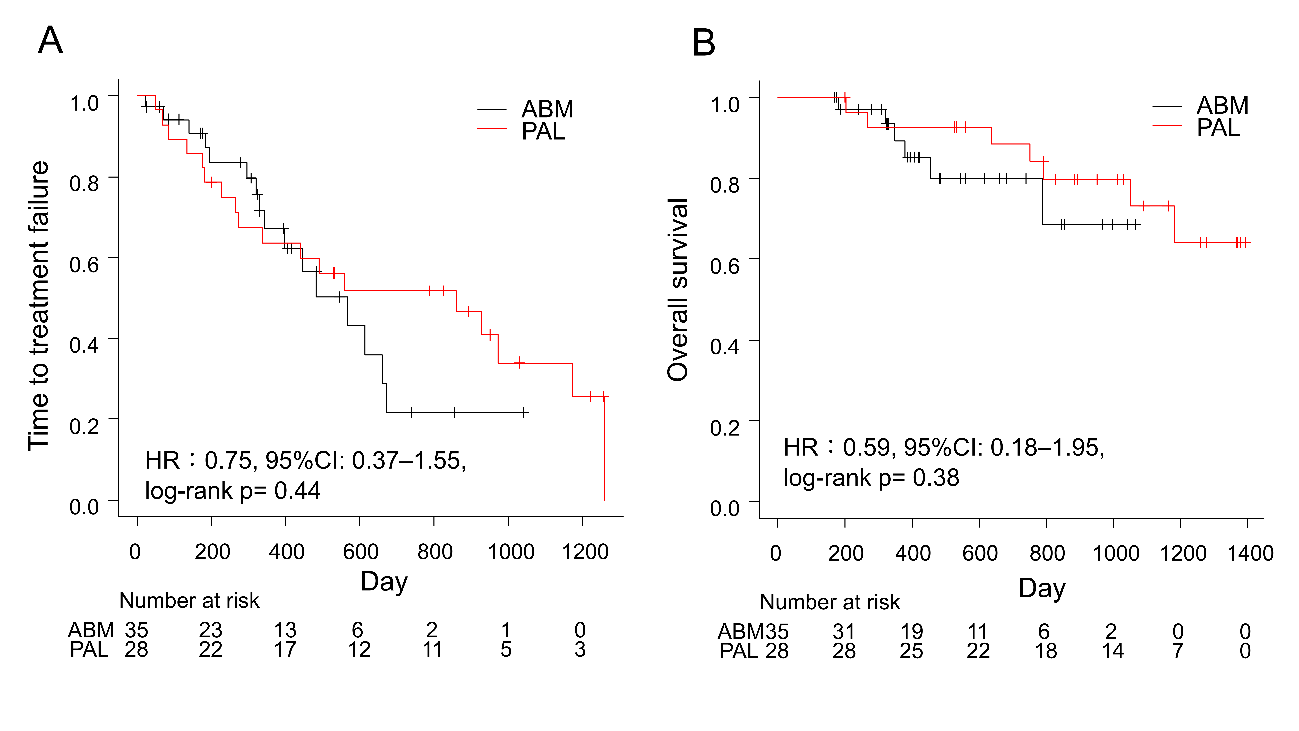


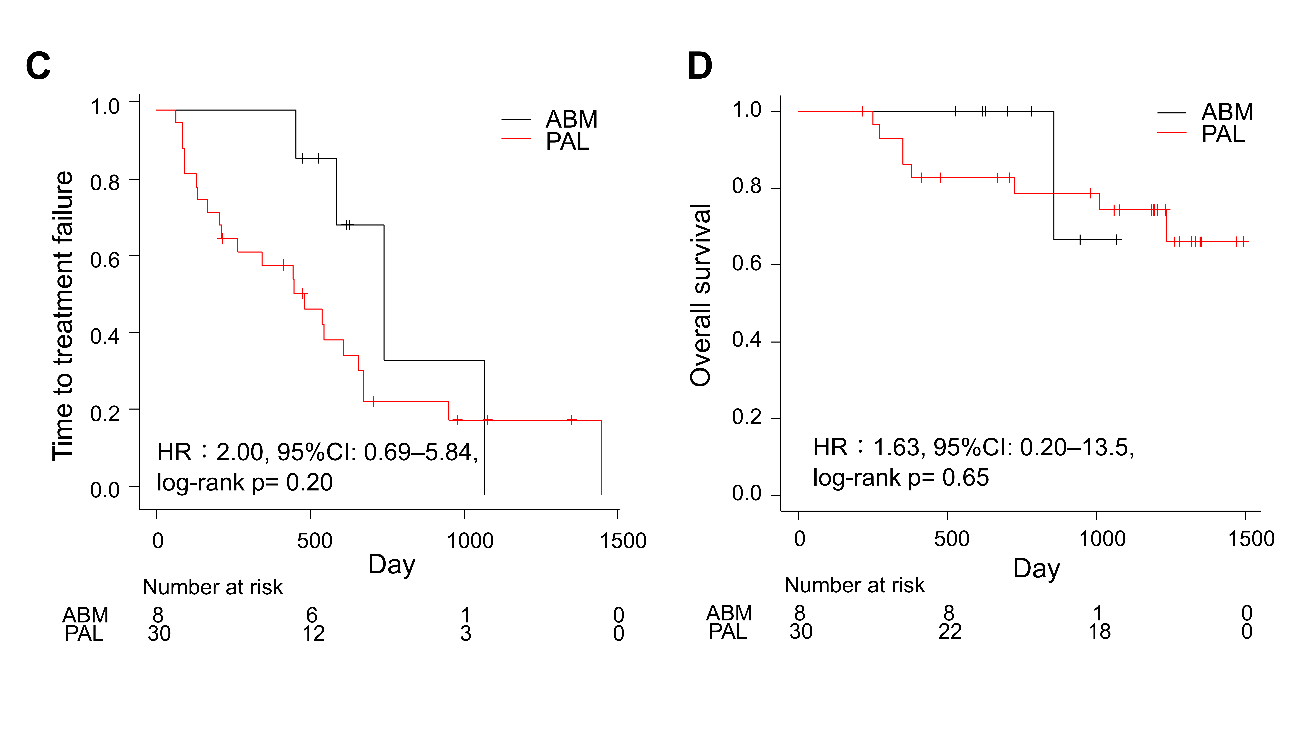


Abbreviations: ABM, abemaciclib; CDK4/6i, cyclin-dependent kinase 4 and 6 inhibitors; CI, confidence interval; HR, hazard ratio; PAL, palbociclib
